# Cultivating leaders for primary health care: A revised approach for transformative development

**DOI:** 10.4102/phcfm.v16i1.4410

**Published:** 2024-04-22

**Authors:** Klaus B. von Pressentin, Angela de Sá, Paddy Pampallis, Tasleem Ras

**Affiliations:** 1 Department of Family, Community and Emergency Care, Faculty of Health Sciences, University of Cape Town, Cape Town, South Africa; 2 Department of Health and Wellness, Western Cape Government, Cape Town, South Africa; 3 Integral Africa: The Coaching Centre, Cape Town, South Africa

**Keywords:** leadership, primary health care, family practice, education, medical, graduate, coaching

## Abstract

Family physicians require leadership skills to strengthen team-based primary care services. Interviews with South African district managers confirmed the need to develop leadership skills in family physicians. The updated national programmatic learning outcomes for South African family physician training were published in 2021. They sparked the need for curriculum renewal at the University of Cape Town’s Division of Family Medicine. A review of the leadership and governance module during registrar training showed that the sessions were perceived to be content heavy with insufficient opportunities for reflection. Following a series of stakeholder engagements, the module convenors co-designed a revised module that was blueprinted on the updated learning outcomes. The module incorporates a group coaching style, facilitating learning through reflection on one’s experiences. The revised module was implemented in 2022. It aims to provide a transformative learning experience centred on students’ perceptions of themselves as leaders, as well as professional identity formation and resilience building. This short report describes preliminary insights from the revised module’s developmental phase and forms part of an ongoing iterative evaluation process.

**Contribution:**

Family physicians should lead across all their defined roles. Formal and informal learning opportunities are needed to facilitate their growth as leaders and help them to meet the health needs of communities served by an evolving health care system. This short report describes an example of a revised postgraduate module on leadership and governance, which may be of value to clinician educators and academic departments exploring innovative methods for the African region.

## Introduction

The political commitment to high-quality primary health care has been renewed several times over the past few decades, both globally and regionally.^1^ South Africa shares this commitment and recognises the role of family physicians (FPs) as members of high-quality primary care teams in its national development plan.^2^

The recent pandemic’s disruptive effect on society and health services is compounded by the rising burnout among health workers.^3,4^ A renewed focus on self-awareness and caring for health workers adds to the growing awareness of the need to shift how health care teams and systems are managed at all levels.^5^ The notion of the complex adaptive system is supported by the need to establish a learning organisational culture and a values-based approach to leadership strategies.^6,7^ These ‘messy realities’ are also manifested at the primary care level, and managers and leaders are grappling with the need for how best to support their teams to improve the quality of services with a shrinking budget in a changing health care landscape.^8^

Family physicians are seen as leaders of interventions to strengthen the district health system (DHS) within their sphere of influence via their teams. Previous research has demonstrated that FPs must develop essential leadership skills to influence their context.^9^ Interviews with district managers revealed that FPs lacking leadership skills or unable to integrate into their clinical teams were deemed ‘at the mercy of the system’. Family physicians with leadership skills and qualities, resilience and the ability to be change agents were seen as able to shape their context. A consequent expectation of FPs in the DHS is that they will help the health care system to improve, expand and develop.^10^

The updated programmatic learning outcomes for training South African FPs were published in 2021.^11^ These learning outcomes define the postgraduate specialist training of FPs across all nine programmes. Unit standard 1 of these learning outcomes describes the leadership and clinical governance skills and capabilities required of graduates.

## The postgraduate programme at the University of Cape Town: A need for curriculum change

The University of Cape Town (UCT) Division of Family Medicine has engaged with these outcomes to revise the training of registrars in this domain. The Division offers postgraduate training at diploma, Masters (MMed) and doctoral levels. Formal training in leadership and governance has been part of the MMed programme for several years and was resumed in 2021 after a 1-year hiatus during the COVID-19 pandemic. At the end of 2021, as part of the annual review with staff and students, the need for curriculum change was identified to align with the updated national learning outcomes. The feedback from the 2021 student cohort demonstrated the need to provide more time for the sessions and opportunities for reflection on how the theory relates to practice, professional identity formation and building resilience.

## A revised curriculum, revisiting educational principles and growing new partnerships in delivering the module

The module was revised in a participatory and engaging manner. A series of engagements with key stakeholders (students, facilitators and external stakeholders) were held at the start of 2022 to review and plan the revised module (content and delivery). We collaborated with a certified leadership coaching centre via one of our joint appointee academic staff members, A.d.S, who was trained via this centre as an accredited coach and joined K.v.P as co-convenor. The revised leadership and governance module was piloted in 2022 and has the following changes as informed by stakeholder engagement:

- The module content was blueprinted on the national programmatic learning outcomes in the domain of leadership and governance (Unit Standard 1), which encompasses sub-domains addressing developing self optimally as a leader, offering leadership within the DHS, leading clinical governance activities in the DHS and influencing DHS corporate governance.^11^
- The module duration was expanded from seven sessions over 4 months in the second semester of year 3 of the MMed programme to 12 sessions spread over both semesters in year 3.
- To help participants discover their leadership personality profile, the Five Lenses Enneagram was employed. This tool explored emotional resilience, personal mastery, social drives (values, building on a developmental view), energy centres (head, heart and gut), and the Enneagram.^12^ This was followed by an initial personal debrief with a certified coach focusing on the first three lenses, which in turn was followed by ongoing group reflections on habitual strategies and styles of being in the world.
- We incorporated a group coaching approach across the 12 sessions to help frame the learning content by linking it to the context of the students to facilitate the transferability of the learning.^13^ The coaching approach fosters self-discovery and uses inquiry to evoke awareness and insight. Here, the coach serves as a thought partner who uses deep questions and close listening to facilitate personal development to sustain change. The need for a positive, supportive process that recognises the difficulties presented by the context in which we work supported our decision to incorporate this coaching approach to enable participants to take a third-person perspective when viewing their roles from the individual and collective perspectives: how we show up and live out our internal drives, how we view and engage with our relationships and organisational culture spaces and how we interpret the manifestations of our explicit organisational system structure.
- The 12 sessions were organised across the unit standards, with four of the 12 sessions focusing on leadership development at the individual and team levels (unit standards 1.1 and 1.2) and the remaining eight sessions focusing on leadership and governance content presented by content experts (unit standards 1.3 – 1.5) followed by a facilitated reflection. The example in Table 1 demonstrates how the 90 min content session by a content expert is complemented by a 90 min facilitated reflection on learning by a coaching expert.
- We invited additional participants from our context to ensure the group size was between 8 and 10. Our third-year registrar cohort usually only consists of three to five students. These additional participants included recent postgraduate diploma graduates and early-career FPs working in the local setting. A mixed group experience was an opportunity to incorporate different experiences into the group coaching sessions.

**TABLE 1:** An example of how the content and coaching experts provided a complementary learning experience for the participants.

Unit standard 1.5: Corporate governance 13:30 – 15:00	Facilitated reflection 15:15 – 16:45
*Facilitator: Content expert* Understand the principles of financial management. Be able to communicate effectively with those responsible for corporate governance.	*Facilitator: Coaching expert* The group could consider scenarios where they have had to address the tension between managers and clinicians regarding cost issues. Possible questions: *How do we manage a team that feels their value is only measured in quantity and not the quality of their work – the eternal stats issue?* *How do we present our arguments for reallocating resources? How do we see ourselves in relation to those who hold the purse strings?*

## Early feedback and navigating the transition: Looking back to plan forward

The class of 2022’s reflections confirmed the module’s value in facilitating self-awareness to ensure wellness and meaning making. The group size (eight participants) and mix of career stages and experiences were seen as valuable in providing a rich learning environment. The participants appreciated the role of the group coaching approach in facilitating learning at individual and collective levels. Some participants also experimented with using the coaching approach in their teams, enabling them to listen more intently and offer leadership to junior colleagues.

As a group of conveners, we will continue to develop this module in the spirit of co-creation by involving current students and recent graduates in the design and updating of the curriculum. We also include students in assisting the convenors in the curriculum evaluation. Growth as a leader beyond the module and programme is a desirable outcome, and we are actively engaged in managing transitions into new roles and contexts. This links well with the SAAFP Next5 initiative, which started in 2021 at a national level to support newly qualified family medicine specialists.^14^

## Lessons learned that may assist regional training programmes in family medicine and primary care

Leadership training and practice in primary care have been dominated by the historic power relationship between the ‘higher’ levels of specialist care provided in secondary and tertiary levels of health care.^15,16^ The dominant discourse is that of specialist care representing expert and superior care, which is a complete departure from the international commitment to primary health care, which has been shown to provide better health outcomes at lower costs to users and governments. The renewed commitment by the South African government to strengthen primary health care via the DHS requires specific educational interventions and leadership development programmes relevant to our setting.^17^ These clinician leaders and their teams face the realities represented by the confluence of the harsh realities of the social determinants of health and the discordant supply of human resources between the public and private sectors, the urban and rural context and the primary and specialist levels of care.^18^

The revised curriculum at the UCT allowed both students and facilitators to bring their lived experiences into the conversation during the group reflections facilitated by the coach. This module engaged participants in a learner-centred manner, allowing them to grow as self-aware clinical leaders capable of supporting transformation in primary care and health service teams. This adult learning style incorporates critical and social learning theories, which underpin reflection and professional identity formation.^19^ The coaching approach includes integral theory for understanding the full spectrum of human consciousness and development, providing a platform for vertical and horizontal leadership development.^20^ We appreciate that being, becoming and growing as a leader is not simple. Box 1 expands on the interpretations of key terms typically associated with leadership practice and development, as shared by the coaching expert who collaborated in revising the module. Guiding conversations allow for transformative learning experiences by disrupting the status quo assessments of self and context. Each cohort can share their growth journey by developing stretch goals and shared accountability in the group.

BOX 1 A reflective piece by one of the module facilitators, Dr Paddy Pampallis.

**The multifaceted endeavour of leadership practice and skills and the need to develop a holistic approach to its development**

Leadership is neither a simple matter nor being a doctor or professional. It cannot be defined as a word as there exist many interpretations. We are trying to surface the many nuances of this role so that the module participants have these in their consciousness. Once conscious, they can attend to them. If not aware, we cannot do anything about them—they operate behind the ‘door’. So, we must distinguish how we understand leadership skills, qualities, praxis, and capabilities. We may see them as different yet linked parts of the whole.

This module forms part of a journey for an inclusive and integral approach to leadership development. There has been a long attempt to shift the model of the doctor and that of medicine and healing from a Western rational, biological system to an integrated system. Introducing an integral approach is an attempt to engage the person, individual, and collective, with a range of interior and exterior lenses onto their leadership so that they can ‘become that’. Integral Africa theory includes our African collective and indigenous healing approaches that expand the ‘body’ (mind, body, heart, spirit, shadow) of medicine along the lines of the good, the true and the beautiful. This engages wisdom philosophy, psychology, spiritual traditions, systems theory, biology, physics, and technology, to name the core ones.

*Leadership skills:* Those overt skills that are usually measured as tangible artefacts or signifiers of a leader’s competence in their domain. These are often contextual, cultural, and professionally driven (or inhibited) by multiple conditions reaching beyond those considered in the role, key performance indicators, or as a value-driven principle. These would already be values articulated by what family medicine describes as linked to vision and purpose, but what people look at in our national context is the skills of doctors as managers for budgeting, running a clinic smoothly, non-adversarial teams, etc. You will typically add these to a curriculum vitae as they are what promotions are made of.

*Leadership qualities:* These qualities extend beyond skills to values (an outer indication of inner awareness and capacity). I have been trying to enable people to see these not just about what we do (while important) but also about who we are. This would refer to humaneness’s inner and outer qualities through service in leadership, roles, and professions. Just as they are asked, who is the doctor? This refers to the doctor or health practitioner relationships, where people have different skills, knowledge, and experience sets but have equally valid considerations, intuitions, and cognitions of their health. We seek to cultivate access to a broader construct of quality and skill that engages the whole person as a leader and health professional and invites them to be perceived for inner qualities beyond a skill. So, in the African sense, personhood is essential, and this goes beyond the individual to their sense of being part of a lineage.

*Leadership capabilities:* Similarly, we have been drawing attention to inner and outer capabilities, including skills and inner and outer capacities. Inner – to manage complexity and tensions; outer – to have space in the physical world to do the work of their profession, role, purpose, leadership, and being. I speak of leadership capacity, availability and potential as necessary for training and development. These can be inner and outer conditions, individual or collective.

All these concepts and qualities are essential. If we make these distinctions, leaders (and those who help them grow) will know what and where to practice.

## Conclusion

We believe that the revised module contributes to a transformed curriculum, allowing for growth and leadership development. These changes also allow for deep and meaningful learning experiences to facilitate resilience and grow a community of learning and practice. The group coaching approach ensures a commitment to support each other, honour the privileged information shared during sessions and allow for a transformative learning experience in a safe environment. Our educational research will inform the MMed module delivery and the development of a planned short course aimed at leaders in primary care teams. We hope our experience may benefit the wider African region as we work together to identify contextually relevant educational strategies to develop future primary care leaders.

We as the authors would like to acknowledge the support from the division and departmental leadership team. We would also like to acknowledge the contribution of the students and recent graduates for their role in cocreating and shaping the revised module. We acknowledge the support of the UCT in awarding the team its Curriculum Change Grant 2023, which was used to deliver and evaluate the revised module.
